# Healthcare Challenges and Future Solutions in Dental Practice: Assessing Oral Antibiotic Resistances by Contemporary Point-Of-Care Approaches

**DOI:** 10.3390/antibiotics9110810

**Published:** 2020-11-14

**Authors:** Georgios N. Belibasakis, Bodil K. Lund, Carina Krüger Weiner, Benita Johannsen, Desirée Baumgartner, Daniel Manoil, Margareta Hultin, Konstantinos Mitsakakis

**Affiliations:** 1 Department of Dental Medicine, Karolinska Institutet, Alfred Nobels allè 8, 14104 Stockholm, Sweden; Bodil.Lund@uib.no (B.K.L.); carina.kruger-weiner@sll.se (C.K.W.); daniel.manoil@ki.se (D.M.); margareta.hultin@ki.se (M.H.); 2 Department of Clinical Dentistry, University of Bergen, 5009 Bergen, Norway; 3 Department of Oral and Maxillofacial Surgery, Haukeland University Hospital, 5021 Bergen, Norway; 4 Department of Oral and Maxillofacial Surgery, Folktandvården Stockholm, Eastman Institutet, 11324 Stockholm, Sweden; 5 Hahn-Schickard, Georges-Koehler-Allee 103, 79110 Freiburg, Germany; Benita.Johannsen@Hahn-Schickard.de; 6 Laboratory for MEMS Applications, IMTEK—Department of Microsystems Engineering, University of Freiburg, Georges-Koehler-Allee 103, 79110 Freiburg, Germany; Desiree.Baumgartner@imtek.uni-freiburg.de

**Keywords:** oral antibiotics, resistance genes, point-of-care, one health, dental practice

## Abstract

Antibiotic resistance poses a global threat, which is being acknowledged at several levels, including research, clinical implementation, regulation, as well as by the World Health Organization. In the field of oral health, however, the issue of antibiotic resistances, as well as of accurate diagnosis, is underrepresented. Oral diseases in general were ranked third in terms of expenditures among the EU-28 member states in 2015. Yet, the diagnosis and patient management of oral infections, in particular, still depend primarily on empiric means. On the contrary, on the global scale, the field of medical infections has more readily adopted the integration of molecular-based systems in the diagnostic, patient management, and antibiotic stewardship workflows. In this perspective review, we emphasize the clinical significance of supporting in the future antibiotic resistance screening in dental practice with novel integrated and point-of-care operating tools that can greatly support the rapid, accurate, and efficient administration of oral antibiotics.

## 1. Clinical Use of Antibiotics in Dentistry 

Oral infections represent the most prevalent chronic diseases, with more than a billion cases globally. In 2015, they amounted to US$ 544.41 billion of direct costs and productivity losses, the highest levels of per capita expenses being observed in high-income North America and Western Europe. Oral diseases, in general, were the third more costly diseases in the EU-28 countries in 2015 [1]. Severe tooth loss accounts for 67% of global productivity losses due to dental diseases, followed by severe periodontitis (21%) and untreated caries (12%) [2]. Periodontitis affects the tissues that surround and support the teeth, whereas dental caries affect dental hard tissues, causing tooth decay. These are the two most prevalent oral diseases, which may lead to progressive loss of the bone and soft tissue attachment, and eventually tooth loss. Untreated, dental caries invariably lead to root canal infections and subsequently acute dentoalveolar abscesses (odontogenic infections) [3], conditions which may warrant the urgent use of antibiotics. There is also evidence that oral infections, such as periodontal diseases, may deteriorate the systemic health status, particularly in the elderly population [4,5].

During previous decades, prophylactic antibiotic prescription was considered imperative for the prevention of infective endocarditis in high-risk patients undergoing invasive dental treatment. Nevertheless, national guidelines among many EU countries concluded on insufficient scientific evidence to support this notion. Antibiotics in dentistry are mainly indicated for treating acute odontogenic infections and as prophylactics preceding invasive procedures. The indication for antibiotic prophylaxis is either a patient with inherent risk for serious infection following treatment or a procedure with an unacceptably high risk for postoperative infection. The treatment of chronic infections is more controversial, and its role in the treatment of periodontitis and peri-implantitis is debated. In Sweden, approximately 6% of antibiotics usage is prescribed due to dental procedures [6]. The most commonly used antibiotics in dental prophylactic and treatment procedures are amoxicillin, metronidazole, and clindamycin. 

## 2. Antibiotic Misuse in Dentistry and Its Relevance to Antibiotic Resistances 

Observations from the Swedish strategic program against antibiotic resistance (STRAMA) point to an overall decrease in antibiotic prescriptions over the last 10–15 years, with a decrease in total prescriptions in dental practices from 35 to 27 per 1000 inhabitants between 2007 and 2016, probably due to the revised prophylaxis guidelines. Yet, significant differences in the prescription patterns were still noticeable between dental practices in greater cities and rural provinces [7]. This trend is also observed in the US, where prescriptions in dental practice accounted for 77.5 per 1000 inhabitants, with a two-fold geographical variability in some instances [8]. Furthermore, a US survey in 2015 revealed that dentists were responsible for more than 2.9 million antibiotic prescriptions, exceeding those of several other medical and allied healthcare provider specialties [9]. This is largely due to the fact that antibiotics are prescribed empirically and contrary to any national guidelines, the underlying reasons being the desire to avoid clinical complications or the tendency to put professional experience before guidelines [10,11]. Dentists prescribe up to 11% of common antibiotics, which varies with respect to world region [12,13,14]. Yet, a large proportion of these prescriptions are deemed unnecessary [15] and may not provide added value to the treatment outcome [16]. Indicatively, up to 80% of antibiotics used in dentistry for prophylaxis is considered inappropriate in the US [17], while in a UK study with General Dental Practitioners, approximately two-thirds of antibiotics were prescribed even though there was no evidence of a spreading infection [18]. National health systems need to streamline their available resources in order to address the frequent antibiotic usage in dental practice, from both global oral health and individual well-being perspectives. 

A single course of antibiotics may cause higher ecological disturbances in the gut, rather than the oral microbiome [19]. Indicatively, clindamycin that is also used in dentistry, has a relatively high risk of gastrointestinal adverse effects, similar for both single doses used for prophylaxis and prolonged courses used for therapeutic reasons [20]. Therefore, antibiotics may not only promote the emergence of resistance mechanisms in previously naïve strains, but also increase the susceptibility of treated patients to colonization by antibiotic-resistant strains and allow the potential outgrowth of already-resistant bacteria commensally present in the individual [10,21]. Thus, antibiotics used for oral infections may also cause ecological adverse effects on other systems of the human body that contribute to the acquisition and development of antibiotic resistances (ABRs). 

## 3. Development of ABRs and Discovery of ABR Genes

ABRs emerge in microbes when they develop the ability to overcome the medicaments that are designed to inactivate them, allowing their continuous activity or growth. This is the most crucial underlying problem of the frequent usage of antibiotics. Infections caused by ABR-carrying microorganisms are recalcitrant to treatment and pose an increasingly serious public health threat. The mechanisms of ABR among bacteria can be intrinsic or acquired [22]. Intrinsic refers to the ability of a bacterium to resist antibiotic action by inherent structural or functional characteristics. This feature is more common among Gram-negative bacteria, attributed to the composition of the wall and membrane structures, the absence of susceptible targets, and eventually the inability of antibiotic agents to cross the outer membrane. Acquired ABR can occur through mutations or horizontal gene transfer and typically results in (i) alteration of the antibiotic molecule, (ii) modification of the antibiotic’s target, (iii) increased antibiotic efflux, or (iv) reduced antibiotic uptake [23]. As an example, β-lactamases able to hydrolyze several β-lactams antibiotics provide multi-resistance towards various molecules of the penicillin, cephalosporin, and carbapenem classes [24].

ABR genes are readily found in the oral microbiome, even in individuals not recently exposed to antibiotics, including humans in isolated indigenous populations, and in commensal bacteria, not only pathogens. This has resulted in a paradigm shift from focusing on the carriage of ABR in pathogenic bacteria to a broader concept of an ‘oral resistome’, which includes all ABR genes present in the oral microbiome [25]. A plausible explanation for this observation is that oral bacteria tend to grow on oral surfaces (e.g., teeth) in the form of multi-species biofilms. The ‘ecological lifestyle’ of the biofilm is an ideal environment for horizontal gene transfer and spread of ABR genes among the constituent bacteria. Hence, bacteria inhabiting the oral cavity are a reservoir of transferable ABR [26]. This exact polymicrobial and diverse etiological nature of oral infections makes it, nevertheless, virtually impossible to identify all existing ABR genes in the plethora of species identified in the oral cavity so far (about 700 species, but possibly >20,000 phylotypes). 

Current methods for the functional identification of novel resistance genes are relatively low-throughput and time-consuming. With the use of Whole-Genome Sequencing (WGS) [27] and metagenomics [28], it is now possible to characterize holistically ABRs, be it on individual species or on a broad microbiome level. WGS screening has been recently applied to the multidrug-resistant opportunistic pathogen *Klebsiella pneumoniae* and was fundamental in determining the transferable colistin resistance gene *mcr-1* as a crucial ABR element in this species [27]. Such analyses have revealed a broad distribution of ABR genes in the oral microbiome, supporting the establishment of the ‘oral resistome’ concept [28]. Monitoring the oral resistome prior to antibiotic administration opens a window for enhancing the efficiency of antibiotic stewardship programs by preventing unjustified or erroneous prescriptions [25], via implementing routine microbial diagnostic pre-evaluation [29].

## 4. Justification of ABR Screening at the Dental Point of Care

Screening ABR at the dental point of care (POC) is an ambitious goal. In order to justify this, it is important to assess in advance the individual needs of the national health system that is about to implement this. As an example, in the Scandinavian region, lack of efficacy of dental treatment due to ABR is still rare, and antibiotic stewardship is equally successful in medicine and dentistry, since the fields have developed side by side for many years. This is also highlighted by the common microbial diagnostic methods and technologies (including sensitivity testing) that are used in hospital laboratories for both medical and dental infections. Yet, this is likely to be a much more common problem in the future, given the increasing life expectancy and increasing number of patients surviving and living with immunocompromising conditions or medication. In this light, the approach proposed in this review can deliver great value for problems of tomorrow’s dental care.

At present, though, the main problem linked to the over-prescription of antibiotics in dentistry is not ABR itself, but lack of knowledge regarding indications, side effects, and contraindications of antibiotic use, as well as refraining from following available guidelines. Hence, we consider that at present, the most relevant usage of POC screening for ABR is in situations where the general dentist needs to perform culturing. These clinical scenarios include patients with: (i) recurrent infections, (ii) failed treatment of an infection, (iii) a compromised immune system (cancer, transplant, high-dose corticosteroids, chemotherapy, radiation therapy, pronounced granulocytopenia, etc.) presenting with dental/oral infections, (iv) early or late signs of severe infection (e.g., infection spreading beyond the oral cavity via the anatomic spatia surrounding it or rapid infection onset and extensive swelling), and (v) patients with severe post-operative infection after oral surgery. In the specialist dental care domain, the use of a POC platform for ABR screening would be mainly relevant in oral and maxillofacial surgery, oral medicine, and pedodontics, particularly in the case of severe infections of dental/oral origin and in case of need for admission to hospital.

## 5. Designing ABR Screening Methods for POC Dental Applications

**Figure 1:**
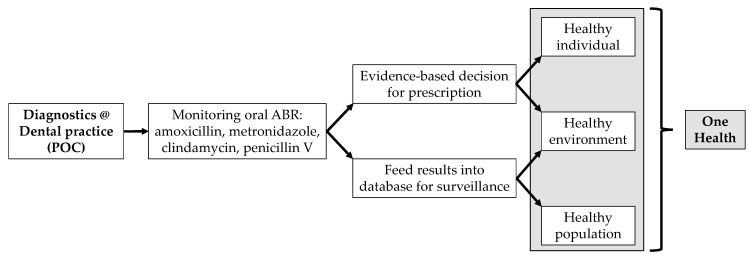
Flow chart indicating the concept of the ‘diagnostic and resistance monitoring hub’ in dental practice, adapting to the One Health solution.

In order to make rapid and evidence-based decisions on selecting or excluding a specific antibiotic during a patient’s visit, there needs to be a ’diagnostic hub’, featuring the necessary tools and being incorporated into the dental practice, i.e., at the POC, instead of a centralized hospital laboratory. This may be achieved by rapid, labor-free, molecular-based analyses at the POC, as opposed to traditional laboratory-based approaches such as cultures [30]. Hence, the utility must operate on a user-friendly interface that facilitates the routine usage by the dental practice care-providers. Moreover, ABR screening at POC in patients warranting dental treatment, or simply during regular oral health check-ups, may allow mapping the distribution of different ABR fingerprints among populations and geographical locations, both nationally and internationally. This can lead consequently to the creation of a surveillance network that can be applicable to the population level and, with the proper use of digital tools, to embrace the issue of oral antibiotic resistances in a One Health holistic approach (Figure 1).

Selection of ABR genes is a preamble for developing assays to detect them rapidly in a POC setting. Screening for suitable ABR genes takes place in oral resistome databases or selected species genomic databases, by using high-end bioinformatics in silico tools. Screening for ABR genes on the sequenced genomic material with contemporary bioinformatics software can be done by platforms such as ResFinder [31], after uploading the WGS data obtained from clinical isolates. The next step after the bioinformatics outcome is the nucleic acid amplification assay design. The detection specificity to the selected resistance genes derives from the corresponding primers and potential probes. TaqMan or SYBR Green quantitative PCR assays are suitable, as they allow the monitoring of several genes of interest simultaneously [32,33]. The development of these assays is typically done in a buffer matrix, in order to avoid inhibitors for the assessment of the assay limit of detection. Nevertheless, in real life, the starting material can be a complex biological matrix, such as saliva, dental plaque, or suppurate, which requires pre-analytical steps [34], as gene amplification directly from the obtained clinical material would lead to reaction inhibition and skewed results. Therefore, nucleic acid extraction and purification techniques have been developed accordingly, some of them operating as column-based, and some based on Boom chemistry [35]. Such biochemical processing steps like extraction, purification, amplification are typically performed in a manual way in a central laboratory and, especially in the case of multiplexing, are prone to pipetting or handling errors even by experienced personnel. Furthermore, the time from sample collection to results may range from hours to days. Such delay is incompatible with the fast decision-making required to select an appropriate antibiotic while the patient is still at the dental practice. Thus, the assays themselves, even when highly sensitive or multiplexed, are, alone, not sufficient to solve the challenge that they are built for.

## 6. Transfer of ABR Assays in Fully Automated POC Platforms for Dental Practice

**Figure 2:**
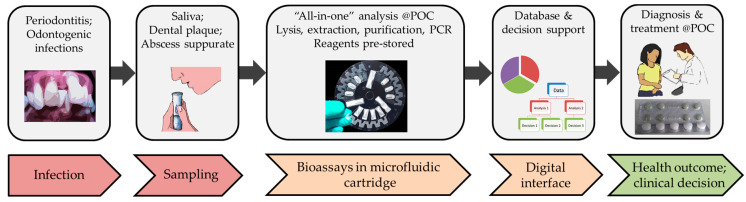
Oral diagnostic and resistance screening workflow at the dental POC.

Promising technological approaches that can play a key role in ABR screening at a dental POC are emerging. These refer to fully integrated and automated systems, which typically make use of microfluidic technologies integrating all biochemical components that are required to perform the process steps that would otherwise be performed in a microbiological laboratory. Such miniaturization and integration can offer several advantages, for example, avoidance of handling errors due to full automation; reduced waste of materials due to the miniaturized nature of the core microsystem; rapid outcome, thereby managing the patients while they are still in the dental practice; option to connect with clinical decision support systems, thereby assisting the dentists in a quick and accurate diagnosis and treatment management (Figure 2).

Nucleic acid amplification-based POC systems have been developed, and some are currently commercially available for application fields like infectious diseases, e.g., respiratory tract, gastrointestinal, sexually transmitted infections. A comprehensive, non-exhaustive list, is summarized by Mitsakakis et al [36]. These systems primarily focus on diagnosis, i.e., specific detection of the pathogen. Some of them do address selective ABR genes, e.g., for methicillin or vancomycin resistance (mecA, vanA, vanB), although in the field of respiratory tract infections encountered in the General Practice, there is still debate as to which ABR genes are the most suitable ones to be detected in combination with the respiratory pathogen species identification [37].

In the dental field, the landscape is inverse. There, the main antibiotic types used are limited to a handful of options, and therefore, the pool of ABR genes that need to be targeted should in principle be more easily defined. However, the POC tools capable of detecting such genes are lacking from the dental settings, mostly because the POC technology has not yet been adopted by the dental practitioners’ community. Only very few dental POC options exist to date, such as the PerioSafe [38]. This, however detects only one protein biomarker, focusing on the immune response, and therefore is not relevant to the issue of ABRs.

**Figure 3:**
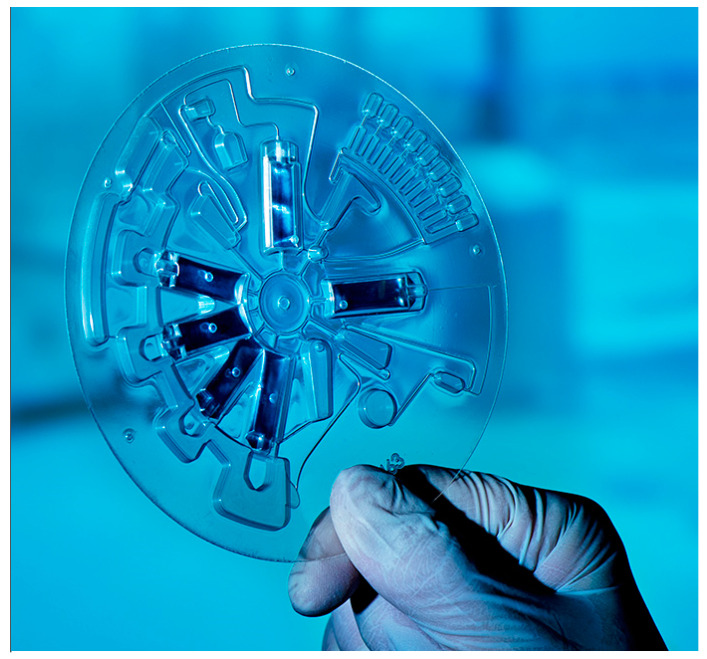
LabDisk cartridge integrating all necessary biochemical components for a fully automated sample-to-answer microbiological analysis. (Figure: Hahn-Schickard, Bernd Müller Fotografie. Reprinted from [44], Copyright (2016), with permission from IOS Press. The publication is available at IOS Press through http://dx.doi.org/10.3233/978-1-61499-653-8-61).

Novel developments of POC platforms have identified niche applications in the dental community. The centrifugal microfluidic platform LabDisk [39] (Figure 3), after having proven its functionality in the field of infectious diseases such as sepsis [40], respiratory tract infections [41,42], and tropical fever [43], in terms of species identification, has been recently implemented in the field of oral health with a dedicated microbiological POC platform for the accurate detection of a panel of oral species associated with dental caries and periodontal disease. The platform consists of a microfluidic disk-shaped cartridge, integrated molecular assays, and a compact, portable disk-processing device, the latter featuring a motor for precise disk positioning, velocity, and acceleration, a thermal unit for thermocycling, and fluorescence detectors for real-time PCR and color multiplexing. The operation is based on saliva samples and in situ processing, purification, and amplification of oral bacteria DNA [44]. The system has been proven to be easily adaptable by changing only the specific primers in the corresponding reaction chambers [43], thereby the conversion from an oral diagnostic to an oral resistance screening tool is easily achievable. It is also worth acknowledging that the beneficial application of such diagnostic tool in dental practices goes along with an implementation strategy package [45]. Such implementation strategy aims to educate and train end-user practitioners to adequately apply and adopt clinically relevant decisions based on microbiological outcomes with utmost priority to patients’ interests. 

Lastly, and in the context of Oral One Health, namely, converging diverse technological fields around the oral health and dental practice, diagnostic screening can work synergistically with (i) cell- and tissue-level research, such as research on stem cells and their microenvironment [46,47]; (ii) materials science and engineering, e.g., in restorative dentistry [48,49]; and digital technologies such as digital panoramic radiographs [50,51]. Such convergence is expected to offer high added value and impact in approaching oral healthcare challenges by making use of diverse contemporary technologies.

## 7. Conclusions

In this perspective review, we considered the clinical importance of antibiotics in the dental practice, when rationally prescribed, but also the related health complications and economic consequences when misused, which still remains too frequent. To tackle with the rapidly evolving challenges of the future, we proposed the enhancement of dental practices with a ‘diagnostic hub’, equipped with rapid molecular diagnosis technologies for the simultaneous identification of several ABR genes directly at the POC. Through the combinatorial use of different primer sets, such POC devices would allow the rapid and reliable detection of multiple clinically relevant ABR genes readily present within the oral microbiome. Hence, ABR testing at the dental POC may have an important medical impact, as it can: (i) replace the current labor-intensive techniques which are practically unsuitable for regular dental examinations, as well as expensive and time-consuming (e.g., several days from sample collection to result reporting); (ii) enhance practitioners’ assessment of the disease by facilitating evidence-based clinical decisions of higher prognostic value; (iii) reduce unnecessary and/or non-efficient antibiotic prescriptions, thereby decreasing the ABR ecological selection pressure; (iv) contribute to personalized monitoring by post-treatment ABR screening; (v) reduce disease complications by shortening the diagnostic period between examination and diagnostic result and preventing side effects of unnecessary and/or un-targeted antibiotic administration; and (vi) increase accessibility to healthcare data by allowing a more widespread collection of epidemiological data on ABR at POC hubs. The POC technologies will enable dental clinicians for the first time to use chair-side applications during a patient’s appointment for the determination of ABRs. With the collection of the suitable material from the site of infection, the clinician will be able to perform a rapid screening of a pre-selected set of ABR genes, performed in a single step by the POC system.
